# Supplementary material for: Reconciling Mining with the Conservation of Cave Biodiversity: A Quantitative Baseline to Help Establish Conservation Priorities
Source: PLoS One. 2016 Dec 20;11(12):e0168348. doi: 10.1371/journal.pone.0168348 (PMC5173368; doi:10.1371/journal.pone.0168348)
Supplement: S1 Dataset — (ZIP) [file pone.0168348.s002.zip › Taxa/Serra Norte/SN_2013/N4E/Lista N4E-55.pdf]

CAVIDADE N4E-0055

Classe	Ordem	Fam/Outros	Gên/Outros	Espécie	S-U	U-S	Zon.
Arachnida	Acari	Penthaleidae		sp.20	X		E
Arachnida	Amblypygi	Phrynidae	*Heterophrynus*	*longicornis*		3	E
Arachnida	Araneae	Barychelidae		jovem	1		E
Arachnida	Araneae	Ctenidae		jovem	1	6	E
Arachnida	Araneae	Nesticidae		jovem		X	E
Arachnida	Araneae	Ochyroceratidae	*Ochyrocera*	sp.3		X	E
Arachnida	Araneae	Ochyroceratidae	*Speocera*	sp.1		X	E
Arachnida	Araneae	Oonopidae	aff. *Xyccarph*	sp.2		X	E
Arachnida	Araneae	Pholcidae	*Leptopholcus*	sp.1	X	X	E
Arachnida	Araneae	Pholcidae	*Mesabolivar*	sp.1	X	X	E
Arachnida	Araneae	Prodidomidae		jovem		X	E
Arachnida	Araneae	Scytodidae	*Scytodes*	*eleonorae*		2	E
Arachnida	Araneae	Segestriidae		jovem	X		E
Arachnida	Araneae	Theridiosomatidae	*Plato*	sp.1	X	X	E
Arachnida	Pseudoscorpiones	Bochicidae		jovem		X	E
Arachnida	Pseudoscorpiones	Chernetidae	*Spelaeochernes*	sp.1	X	X	E
Arachnida	Pseudoscorpiones	Chthoniidae	*Pseudochthonius*	sp.1		X	E
Chilopoda	Geophilomorpha	Schendylidae		sp.1		1	E
Chilopoda	Scolopendromorpha	Scolopocryptopidae	*Tidops*	sp.1	1	2	E
Diplopoda	Polydesmida	Fuhrmannodesmidae		sp.1		X	E
Entognatha	Collembola	Isotomidae		sp.1		X	E
Entognatha	Collembola	Paronellidae		sp.1	X	X	E
Entognatha	Collembola	Paronellidae		sp.4		X	E
Entognatha	Diplura	Campodeidae		sp.1	X	X	E
Insecta	Blattodea			jovem	1		E
Insecta	Coleoptera	Scarabaeidae		sp.2	X		E
Insecta	Diptera	Chironomidae		sp.	X		E
Insecta	Diptera	Dolichopodidae		sp.	X		E
Insecta	Diptera	Phoridae	Metopininae	sp.		X	E
Insecta	Diptera	Psychodidae	Phlebotominae	sp.	X	X	E
Insecta	Diptera	Sciaridae		sp.		X	E
Insecta	Diptera	Tipulidae	Tipulinae	sp.	X	X	E
Insecta	Heteroptera	Reduviidae	Emesinae	sp.	1		E
Insecta	Heteroptera	Reduviidae	Reduviinae	sp.	1		E
Insecta	Homoptera	Cixiidae		jovem		X	E
Insecta	Hymenoptera	Formicidae	*Camponotus*	sp.1	X		E
Insecta	Hymenoptera	Formicidae	*Camponotus*	sp.2	X		E
Insecta	Hymenoptera	Formicidae	*Eurhopalothrix*	sp.1	X		E
Insecta	Hymenoptera	Formicidae	*Eurhopalothrix*	sp.2		X	E
Insecta	Hymenoptera	Formicidae	*Pachycondyla*	sp.1	X		E
Insecta	Hymenoptera	Formicidae	*Pheidole*	sp.2		X	E
Insecta	Hymenoptera	Formicidae	*Pheidole*	sp.3		X	E
Insecta	Hymenoptera	Formicidae	*Solenopsis*	sp.1		X	E
Insecta	Isoptera	Termitidae	*Nasutitermes*	sp.	X	X	E
Insecta	Lepidoptera	Noctuoidea		sp.5		1	E
Insecta	Orthoptera	Phalangopsidae	*Paraclodes*	jovem	6	1	E
Insecta	Orthoptera	Phalangopsidae	*Phalangopsis*	sp.		22	E
Gastropoda	Pulmonata	Bulimulidae	*Naesiotus*	sp.		X	E
Gastropoda	Pulmonata	Subulinidae		sp.5		X	E

Gastropoda	Pulmonata	Systrophiidae	*Happia*	sp.		X	E
Pauropoda	Tetramerocerata			sp.	X		E
Symphyla		Scutigerellidae		sp.		X	E
Amphibia	Anura	Strabomantidae	*Pristimantis*	cf. *fenestratus*	8		E
Mammalia	Chiroptera	Furipteridae	*Furipterus*	*horrens*	1		E
Mammalia	Chiroptera	Phyllostomidae	*Micronycteris*	*microtis*	1	6	E
